# *In Vivo* Effects of Free Form Astaxanthin Powder on Anti-Oxidation and Lipid Metabolism with High-Cholesterol Diet

**DOI:** 10.1371/journal.pone.0134733

**Published:** 2015-08-11

**Authors:** Yung-Yi Chen, Pei-Chi Lee, Yi-Long Wu, Li-Yun Liu

**Affiliations:** 1 Department of Immunity and Infection, Liver research unit, University of Birmingham, Birmingham, United Kingdom; 2 Department of Food Science, Nutrition and Nutraceutical Biotechnology, Shih Chien University, Taipei, Taiwan; 3 Research & development department, Bioptik Technologies Inc. Hsin-Tzu Science Park, Hsin-Tzu, Taiwan; University of Basque Country, SPAIN

## Abstract

Astaxanthin extracted from *Pomacea canaliculata* eggs was made into free-form astaxanthin powder (FFAP) and its effects on lipid metabolism, liver function, antioxidants activities and astaxanthin absorption rate were investigated. 45 hamsters were split into 5 groups and fed with normal diet, high-cholesterol control (0.2% cholesterol), 1.6FFAP (control+1.6% FFAP), 3.2FFAP (control+3.2% FFAP) and 8.0FFAP (control+8.0% FFAP), respectively, for 6 weeks. FFAP diets significantly decreased the liver total cholesterol, triglyceride levels and increased liver fatty acids (C20:5n3; C22:6n3) compositions. It decreased plasma alanine aminotransferase and aspartate aminotransferase. In terms of anti-oxidative activities, we found 8.0 FFAP diet significantly decreased plasma and liver malonaldehyde (4.96±1.96 μg TEP eq./mL and 1.56±0.38 μg TEP eq./g liver) and liver 8-isoprostane levels (41.48±13.69 μg 8-ISOP/g liver). On the other hand, it significantly increased liver catalase activity (149.10±10.76 μmol/min/g liver), Vitamin C (2082.97±142.23 μg/g liver), Vitamin E (411.32±81.67 μg/g liver) contents, and glutathione levels (2.13±0.42 mg GSH eq./g liver). Furthermore, 80% of astaxanthin absorption rates in all FFAP diet groups suggest FFAP is an effective form in astaxanthin absorption. Finally, astaxanthin was found to re-distribute to the liver and eyes in a dose dependent manner. Taken together, our results suggested that the appropriate addition of FFAP into high cholesterol diets increases liver anti-oxidative activity and reduces the concentration of lipid peroxidase and therefore, it may be beneficial as a material in developing healthy food.

## Introduction

Free radicals are oxidative products produced during metabolic processes. It is responsible for modulation of cell growth and signal transduction between cells[1]. Under normal physiological conditions, the excessive free radicals produced in the body can be removed by internal antioxidants and anti-oxidative systems. Insufficient antioxidants in the body can cause damage to health, such as protein degeneration, cell and tissue injury, atherosclerosis, induce cardiovascular diseases, cancer, and promote the aging process[2][3]. Therefore, maintaining the dynamic balance of free radicals and anti-oxidative system in the body is very important. Natural antioxidants not only prevent cell damage caused by free radicals, but also enhance the immune response[4]. Carotenoids are natural antioxidants, with more than 600 known types to date. They can be split into two categories, xanthophyll and carotenes. Astaxanthin (3,3’-dihydroxy-β,β-carotene-4,4’dione, C_40_H_52_O_4_, ASTA) is a xanthophyll carotenoid which is found in many microorganisms and marine animals, such as shrimp, crayfish, crustaceans, salmon, trout, krill, microalgae as well as yeast; and it’s believed to be the most widely distributed lutein in the biosphere. It is a fat-soluble pigment, which plays a pivotal role in maintaining physiological functions. The structure of ASTA consists of two terminal rings joined by a polyene chain[5], which has two asymmetric carbons located at the 3, 3’ position of the β-ionone ring with hydroxyl functional group on each end of the molecule and this increases it ability in capturing per-oxidants[6]. It has been shown to have many biological functions, including anti-oxidative effects, anti-lipid peroxidation[7], anti-inflammatory properties and enhancement of the immune response[7][8][9]. Researches has suggested that the anti-oxidative properties of ASTA can contribute to the prevention of cancer, diabetes and cardiovascular diseases [8][9]. Furthermore, ASTA has a higher anti-oxidative activity on cell membrane, compared to β-carotene and it is a super antioxidant with an anti-oxidative activity that is higher than most of the other antioxidants, including vitamin C, vitamin E, β-carotene, lutein, lycopene, and other catechins[9]. Therefore, in recent years, ASTA has been widely used as a material in the food industry[10].

*Pomacae canaliculata* is a type of large snail, which was originally found in South America. In 2004, research by Dreon MS *et al*., suggested *Pomacae canaliculata* eggs to be rich in ASTA isoforms, which possess high anti-oxidative activity, and is able to inhibit the oxidation response in liver microsomes[11]. Given that *Pomacae canaliculata* eggs contain a high concentration of ASTA, they may be a valuable source in developing ASTA related products in the health food industry. ASTA easily loses its anti-oxidative activity when exposed to heat and light, therefore in this study, the free-form ASTA extracted from the *Pomacae canaliculata* eggs was packaged with mung bean powder and made into free-form ASTA powder (FFAP). The anti-oxidative activities and its effects on lipid metabolism were investigated.

## Materials and Methods

### Astaxanthin extraction and the production of free-form astaxanthin powder diets

ASTA and FFAP (S1 Fig) used in this study were produced and provided by Bioptik Technology Inc (Taiwan). ASTA extraction was performed according to a method described in the US patent US8030523B2, filed by Bioptik Technology Inc. Briefly, *Pomacae canaliculata* eggs were mixed proportionally with deionized water, homogenized (POLYTRON PT-2100, Bestway International Corporation, Taiwan), and the egg shells were removed to obtain a glycoprotein carotenoid solution. Then the protein, sugar, and lipid in the glycoprotein carotenoid solution were removed sequentially to obtain the carotenoid solution. Finally, the pure ASTA was extracted using 95% ethanol and the purity of ASTA was measured using the method described by Skrede *et al* (1986). The purity of the ASTA used in this study was >99.9%. Then, the extracted ASTA was mixed with mung bean powder using a specialized emulsification technique and made into FFAP. The final concentration of ASTA within the FFAP is 1%. The general composition of FFAP was analyzed using the AACC method[12]. Finally, the FFAP was used as a material to produce 3 different FFAP diets in this study (1.6FFAP, 3.2FFAP and 8.0 FFAP; S1B Fig) according to the diet formula shown in Table 1. The doses of ASTA of the 3 FFAP diets are equivalent to 8mg/kg/day, 16mg/kg/day and 40mg/kg/day, respectively. The FFAP diets chosen for this study were based on the ASTA concentrations previous published in animal experiments and clinical studies, which have demonstrated significant beneficial effects of the ASTA in, for example, controlling blood pressure (5mg/kg/day and 50mg/kg/day), lowering liver TG level (30mg/kg/day), reducing liver metastasis (1mg/kg/day), modulating glucose levels (50mg/kg/day) and weight loss (30mg/kg/day) [13–23]

**Table 1:** Diet formula.

Components (100g)	Normal	Control	1.6 FFAP	3.2 FFAP	8.0 FFAP
**Corn starch (g)**	42.5	42.5	40.9	39.30	34.5
**Casein (g)**	25	25	25	25	25
**Sucrose (g)**	10	10	10	10	10
**Cellulose (g)**	6	6	6	6	6
**Soybean oil (g)**	6.67	6.67	6.67	6.67	6.67
**Lard (g)**	3.33	3.33	3.33	3.33	3.33
**Vitamin mix (g)**	1	1	1	1	1
**Mineral mix (g)**	3	3	3	3	3
**Choline bitartrate (g)**	0.25	0.25	0.25	0.25	0.25
**FFAP (g)**	-	-	**1.6**	**3.2**	**8**
**CMC (g)**	2	2	2	2	2
**Total calorie (Kcal)**	400	400	400	400	400
**Total (%)**	100	100	100	100	100
**Cholesterol (g)**		0.2	0.2	0.2	0.2

Normal: Normal diet; Control: Normal diet + 0.2% cholesterol; 1.6FFAP: control diet + 1.6%FFAP; 3.2FFAP: control diet + 3.2% FFAP; 8.0FFAP: control diet + 8.0%FFAP.

### Animal and ethics statement

45 male Golden Syrian hamsters, *Mesocricetus auratus*, (8-week old) were purchased from the National Laboratory Animal Centre (NLAC, Taipei, Taiwan). The hamsters were acclimatized and housed in a standard animal house with 24 hour air-conditioning at 22±2°C, 12 hr. dark/light cycle and permitted free access to water and food (MGF, Oriental Yeast Co., LTD) for 2 weeks. The animals were monitored daily during the study. The protocols for the animal experiments in this study were approved by the Institutional Animal Care and Use Committee (IACUC), Shih Chien University and conducted according to the guidelines laid down by the IACUC.

### *In vivo* Animal study

Forty five 10-week old male Golden Syrian hamsters (*Mesocricetus auratus*) purchased from the National Laboratory Animal Centre were randomly split into 5 groups, each group contained 9 hamsters, and were fed with 5 different diets, including: normal diet, high cholesterol control diet, 1.6% FFAP diet, 3.2% FFAP diet and 8.0 FFAP diet for 6 weeks. During the experimental period, animals were permitted free water and food, according to *ad libitum* method. Water was replaced daily and the water intake was recorded. The corks were sterilized everyday in an 80°C water bath for 10 minutes. All animals were checked for changes in body weight once a week. Food consumption, water intake, bowel movements and body weight changes were also recorded. Total ASTA intake was calculated using the formula shown below:

Total ASTA intake (g) = Food intake (g) x FFAP (%) x 1% (ASTA concentration in FFAP)

### Sample collections

At the end of the 6-week experiment period, all animal were fasted for 12 hours before being sacrificed, and the liver, kidney, epididymis, heart, lung and eyes harvested from each animal, weighed, snap frozen and stored at -80°C for future analysis. Blood samples were taken by cardio-puncture and collected in heparin tubes. Plasma was obtained by centrifuging blood sample at 3000*rpm* for 10 minutes at 4°C. The supernatant was then collected and stored at -20°C. Fecal samples were collected 3 days before the end of experiment and stored at -20°C for future analysis.

### Biochemical analysis

The TG and TC of each sample were examined enzymatically using commercialized kits, Triglycerides-GPO-PAP (Fortress diagnostics, BXC0272C, UK) and Cholesterol e.p. (liquid) assay (Randox, CH7945, UK), respectively. HDL-C and LDL-C were analyzed using Fortress HDL Cholesterol Precipitant Mono liquid kit (Fortress diagnostics, BXC0422A). The results of each analysis for TC, TG, HDL-C and LDL-C were determined by reading the absorbance at 500nm wavelength. ALT (Alanine aminotransferase) and AST (Asparate aminotransferase) were tested using Alanine aminotransferase kit and Asparate aminotransferase kit (Randox Laboratories Limited, UK) and determined by reading the absorbance at 454nm wavelength using a plate reader. Liver and plasma Trolox equivalent antioxidant capacity (TEAC) were determined according to ABTS radical cation (ABTS^+^) decolorization assay[24] by mixing 10μl sample (tissue homogenate) with 1ml of ABTS^+^ (2, 2’-azino-bis (3-ethylbenzothiazoline-6-sulphonic acid)) solution for 6 minutes in the dark and the anti-oxidative capacity was determined at 734nm wavelength. Vitamin C concentration was tested based on the method from Jagota and Dani *et al*., by mixing sample and Folin-Ciocalteius reagent for 10 minutes. The concentration of Vitamin C was then determined at wavelength of 760nm[25]. Vitamin E was tested using HPLC according to Miller, K.W. *et al*., protocol[26]. Plasma and liver MDA (malondialdehyde) was tested using the thiobarbituric acid reactive substance (TBARS) principle with the result determined at 533nm absorbance[27]. Finally, 8-isoprostane (8-ISOP), catalase (CAT) and superoxide dismutase (SOD) was tested using ELISA kits (8-Isoprostane EIA kit, Catalase Assay kit and Superoxide Dismutase Assay kit) purchased from Cayman Chemical (Michigan, USA) according to manufacturing protocols. Glutathione (GSH) concentration was determined by mixing 100μl sample with 900μl phosphate-EDTA and 100μl *ο*-phthaladehyde for 15 minutes in dark, at room temperature[28]. The result was recorded at Ex350nm/Em420nm using a fluorescent spectrophotometer.

### Liver fatty acid composition

Liver fatty acid composition was tested using gas chromatography (GC) based on the protocol published by Del Rio, J. C. *et al*[29]. Briefly, 3ml liver extract was mixed with 1.5ml ethanol and 500μl of 25% TAMH (Tetramethyl ammonium hydroxide), incubated for 10 minutes before addition of 2ml dH_2_O. Finally, the anhydrous sodium sulfate was collected, from which 2μl was analyzed in a gas chromatography, GC. The fatty acid composition was analyzed by calculating the integral area and retention time.

### Liver reducing power

The liver reducing power was tested based on the protocol published by Oyaizu, M. *et al*., (1986). 100μl sample was mixed with 100μl of PBS (0.2M, pH6.6) and 500μl of 1% K_3_Fe(CN)_6_, and incubated in 50°C water bath for 20 minutes. 500μl of 10% TCA was then added, followed by centrifuging at 3000 *rpm* for 10 minutes. The supernatant was collected and incubated with 500μl deionized water and 100μl of 1% FeCl_3_•6H_2_O for 10 minutes. Finally, the result was recorded by a spectrophotometer at 700nm wavelength.

### ASTA distribution

Tissues, including heart, kidney, lung, epididymis, liver, eyes, and faecal sample were collected as described above. The amount of ASTA in each sample was measured according to Kang, C. K. *et al*., using a high-performance liquid chromatography, HPLC, by passing the sample through Phenomenex C18 Reverse phase column (4.6mm x 250mm, 5μm). Mobile phase: [Methanol: Acetonitrile: Chloroform = 47:47:6 (v/v/v)] at 1.0 mL/min flow rate at 25°C. Then the data was recorded at 474nm wavelength[30]. The content of ASTA was calculated using the integral area and retention time of each sample.

### Statistics

All data in this study was tested using Duncan’s multiple range tests, which provides significance levels for the difference between any pair of means. Pearson correlation coefficient was used to study the correlations between plasma and liver MDA and its anti-oxidant activities. Significance between groups (*p*<0.05) is denoted by dissimilar lowercase letters.

## Results

### Feed efficiency

**Fig 1:**
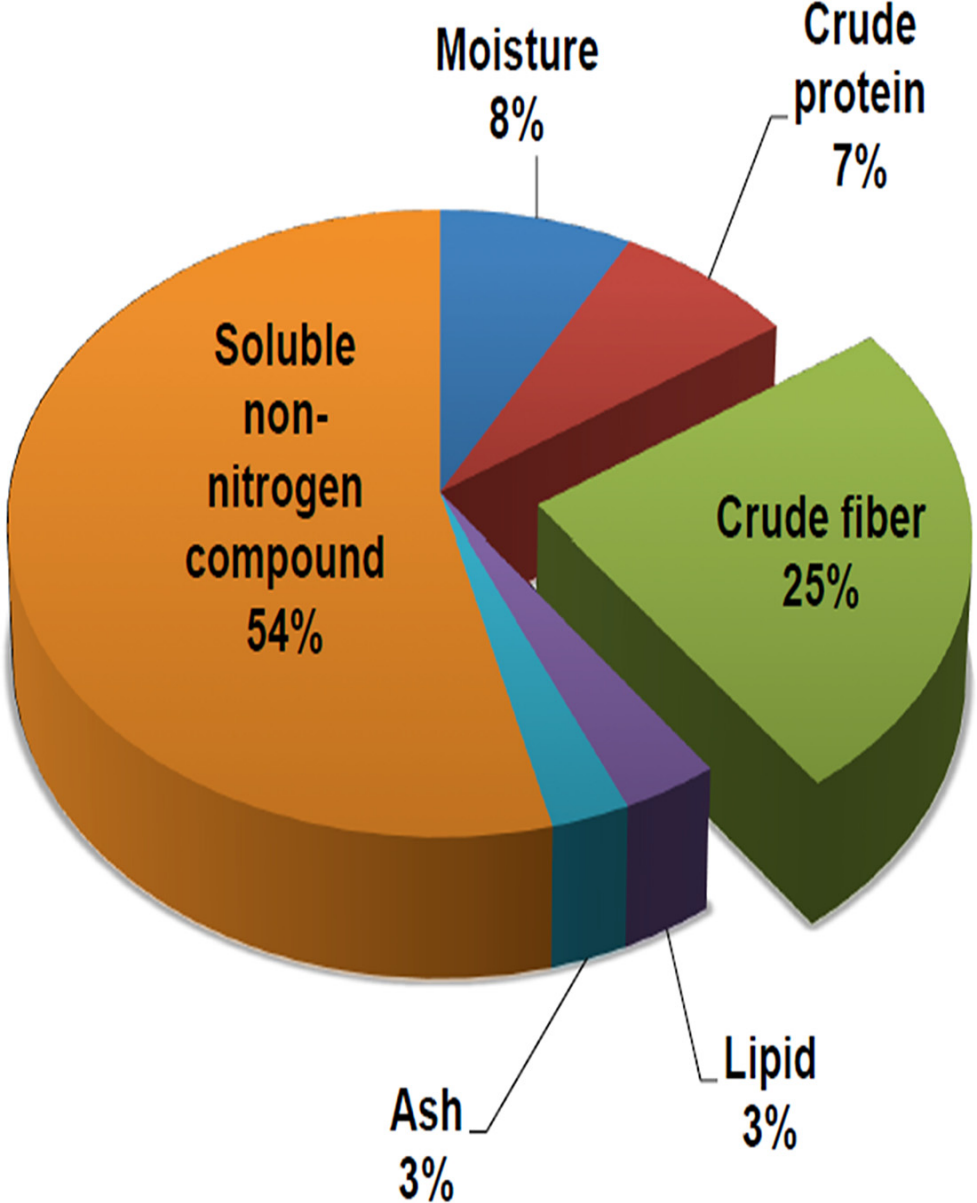
The general compositions of the FFAP. The pie chart represents the general compositions of FFAP. This form of astaxanthin contains large amount of crude fiber and therefore can be used as a source of high fiber food.

Astaxanthin is a type of lipid-soluble substance, which consists of 2 forms, the free form and the ester form. The free form ASTA is easier for digestion and absorption; however, it easily loses its anti-oxidative activity when exposed to heat and light. In order to preserve its anti-oxidative functions, free-form ASTA was made into free form astaxanthin powder (FFAP) by mixing ASTA with mung bean powder. The FFAP was analyzed for ASTA content using HPLC and its general composition was analyzed according to the AACC method (Fig 1). The diet formula for each experimental group, including normal diet, high cholesterol diet, 1.6 FFAP, 3.2 FFAP and 8.0 FFAP diet is shown in Table 1. To investigate the effect of FFAP on feed efficiency, food intake, water intake and body weight changes were recorded. The food intake and body weight changes of the hamsters during the 6 weeks experiment periods were recorded and shown in S2 Fig. Overall, there were no significant differences in these factors between each diet group. The feed efficiency was calculated using the formula: *Body weight changes (g) / Food intake (g) x 100%*. From the results, we found that the normal diet had the highest feed efficiency, at 6.40±1.42%; and the high cholesterol diet had the lowest feed efficiency, at 5.28±1.38% diet. When 1.6 FFAP, 3.2 FFAP and 8.0 FFAP were added into high cholesterol diet, the feed efficiency increased from 5.28±1.38% to 5.69±0.73%, 5.70±2.63% and 6.06±1.76%, respectively. However, there were no significant differences between each group, Table 2.

**Table 2:** The feed efficiency (%) of all test groups.

Group	Feed efficiency (%)
**Normal**	6.40 ± 1.42 ^a^
**Control**	5.28 ± 1.38 ^a^
**1.6FFAP**	5.69 ± 0.73 ^a^
**3.2FFAP**	5.70 ± 2.63 ^a^
**8.0FFAP**	6.06 ± 1.76 ^a^

All values are Mean ± SD, n = 9. All data was tested using Duncan’s multiple range tests, where significance between groups (p<0.05) is denoted by dissimilar lowercase letters. Normal: Normal diet; Control: Normal diet + 0.2% cholesterol; 1.6FFAP: control diet + 1.6%FFAP; 3.2FFAP: control diet + 3.2% FFAP; 8.0FFAP: control diet + 8.0%FFAP.

### Plasma and liver lipid concentrations and liver fatty acid compositions

To study the effect of FFAP diets on lipid changes in the body, hamster organ-coefficient was monitored, Table 3. These data showed that the FFAP diets, including 1.6 FFAP, 3.2 FFAP and 8.0 FFAP, significantly decreased liver weights (3.41±0.13g, 3.40±0.21g and 3.40±0.1g, respectively) and epididymal adipose levels (1.9±0.1g, 8.0 FFAP diet). All data are mean ± SD, *p* < 0.05, compared to high cholesterol control, n = 9 (Duncan’s multiple range tests). In addition, total cholesterol (TC) and triglycerides (TG) in the plasma and liver were screened to study the effects of FFAP diets on plasma and liver lipids changes. As expected, the high cholesterol diet significantly increased plasma TC (90.69±2.69 mg/dL) and TG (60.31±4.76 mg/dL) levels; as well as liver TC (56.1±0.48 mg/g) and TG levels (87.3±0.45 mg/g) compared to normal diet. The addition of FFAP into high cholesterol diets significantly decreased the levels of liver TC (41.4±0.15 mg/g liver, 8.0 FFAP) and TG (69.6±0.18 mg/g liver, 3.2 FFAP and 71.1±0.31 mg/g liver, 8.0 FFAP). All data are mean ± SD, *p*<0.05, compared to high cholesterol control, n = 9 (Duncan’s multiple range tests). However, there was no significant reduction in the levels of plasma TC and TG, Table 4. In addition to TC and TG, the plasma HDL-C and LDL-C values were also studied. The ratio between LDL-C and HDL-C were used as an index of liver lipid metabolism to reflect the risk for atherosclerosis, Table 4. The result showed that despite the concentration of HDL-C and LDL-C in FFAP diets and high cholesterol controls being significantly higher than normal diet, the LDL-C to HDL-C ratio remained stable. This data supports the studies by Iwamoto, T. *et al*., (2000) and Augusti P. R. *et al*., (2012), which also found ASTA diet has no effect on the levels of total cholesterol and the liver lipid metabolisms[31, 32].

**Table 3:** The organ coefficients (%) of all test groups.

Group	Liver (g)	Liver / body weight (%)	Epididymal adiopse (g)	Epididymal adipose / body weight (%)
**Normal**	3.03 ± 0.08 ^c^	2.60 ± 0.10 ^c^	2.18 ± 0.16^ab^	1.85 ± 0.26 ^a^
**Control**	3.96 ± 0.18 ^a^	3.38 ± 0.14 ^a^	2.49 ± 0.18^ab^	2.16 ± 0.13 ^a^
**1.6FFAP**	3.41 ± 0.13 ^b^	3.00 ± 0.16 ^b^	2.00 ± 0.11^c^	1.79 ± 0.18 ^b^
**3.2FFAP**	3.40 ± 0.21 ^b^	2.95 ± 0.12 ^b^	1.99 ± 0.12^c^	1.71 ± 0.22 ^b^
**8.0FFAP**	3.40 ± 0.10 ^b^	3.13 ± 0.19 ^b^	1.90 ± 0.10 ^c^	1.71 ± 0.18 ^b^

Result suggested that FFAP diets significantly decreased liver weights (3.41±0.13g, 3.40±0.21g and 3.40±0.1g, respectively) and epididymal adipose levels. All values are Mean ± SD, n = 9. All data was tested using Duncan’s multiple range tests, where significance between groups (p<0.05) is denoted by dissimilar lowercase letters. Normal: Normal diet; Control: Normal diet + 0.2% cholesterol; 1.6FFAP: control diet + 1.6%FFAP; 3.2FFAP: control diet + 3.2% FFAP; 8.0FFAP: control diet + 8.0%FFAP.

**Table 4:** Plasma and liver lipid concentrations in all test group.

	Blood					Liver	
Group	TG (mg/dL)	TC (mg/dL)	HDL-C (mg/dL)	LDL-C (mg/dL)	LDL/HDL-C ratio	TC (mg/g)	TG (mg/g)
**Normal**	51.64 ± 3.20 ^b^	74.65 ± 3.45 ^b^	25.3 ± 1.65 ^b^	47.3 ± 3.76 ^b^	1.89 ± 0.14 ^a^	45.0 ± 0.21^ab^	75.2 ± 0.37^ab^
**Control**	60.31 ± 4.76 ^a^	90.69 ± 2.69 ^a^	30.19 ± 2.03 ^a^	58.93 ± 2.69 ^a^	1.94 ± 0.14 ^a^	56.1 ± 0.48 ^a^	87.3 ± 0.45 ^a^
**1.6FFAP**	56.60 ± 7.91^ab^	90.87 ± 3.58 ^a^	31.12 ± 2.24 ^a^	61.87 ± 4.25 ^a^	1.99 ± 0.22 ^a^	45.3 ± 0.22^ab^	75.0 ± 0.30^ab^
**3.2FFAP**	60.08 ± 6.86 ^a^	90.82 ± 3.52 ^a^	31.14 ± 2.16 ^a^	60.97 ± 4.21 ^a^	1.95 ± 0.17 ^a^	45.1 ± 0.28^ab^	71.1 ± 0.31 ^b^
**8.0FFAP**	63.37 ± 6.26 ^a^	90.27 ± 3.64 ^a^	32.07 ± 2.44 ^a^	60.71 ± 2.88 ^a^	1.91 ± 0.25 ^a^	41.4 ± 0.15 ^b^	69.6 ± 0.18 ^b^

The result showed that FFAP diet has no effect on plasma lipid levels. On the other hand, FFAP diet significantly decreased liver TC and TG levels, p<0.05. All values are Mean ± SD, n = 9. All data was tested using Duncan’s multiple range tests, where significance between groups (p<0.05) is denoted by dissimilar lowercase letters. Normal: Normal diet; Control: Normal diet + 0.2% cholesterol; 1.6FFAP: control diet + 1.6%FFAP; 3.2FFAP: control diet + 3.2% FFAP; 8.0FFAP: control diet + 8.0%FFAP.

**Fig 2:**
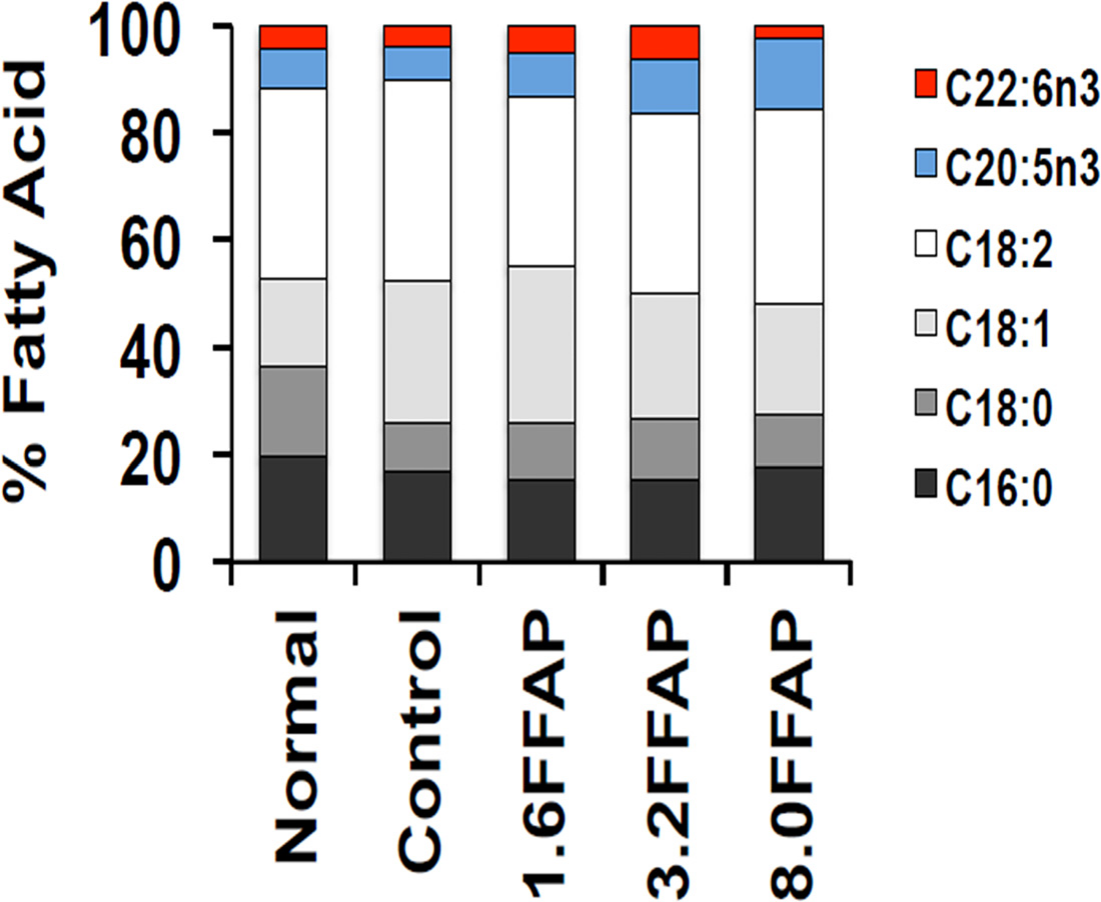
The fatty acid composition in the liver of test groups. The unsaturated fatty acids EPA (C20:5n3) and DHA (C22:6n3) are shown in blue and red. FFAP diets increased the EPA and DHA ratios in the liver fatty acid composition.

In terms of liver fatty acid composition, we noticed that the percentages of EPA (C20:5n3) and DHA (C22:6n3) in the liver fatty acid compositions increased when animals were fed with FFAP diets, compared with controls (Fig 2). From the result, we can see that the liver EPA (C20:5n3) and DHA (C22:6n3) were at 7.55% and 4.28%, when animals were fed with normal control diet; and the percentage decreased to 6.29% and 3.77% respectively when hamsters were fed with high cholesterol control diet. The percentages of EPA and DHA in the liver fatty acid composition increased by nearly 2 fold (EPA: 8.09%, 10.04% and 13.47%; DHA: 5.20%, 6.21% and 2.35%, respectively), after feeding with 1.6 FFAP, 3.2 FFAP and 8.0 FFAP diets, although these changes were not significant.

### Liver functions

Liver function was studied by monitoring the levels of plasma and liver alanine transaminase (ALT) and asparate aminotransferase (AST). High cholesterol diets significantly increased plasma ALT (21.83±4.28U/L) and AST (13.73±5.29U/L) as well as liver AST (data not shown) when compared to the normal control. Interestingly, when FFAP was supplemented into the liver, a significant reduction in the levels of plasma ALT and AST (reduced by 50%-80%) was observed, Table 5. Despite the significant decrease in the levels of liver function indexes (plasma ALT and AST; liver AST) in the FFAP diet groups compared to the normal diet group, the livers gross appearance and the H&E staining in the FFAP diet groups were similar to those in normal control, S3 Fig. Similar to a study by Shen, M. *et al*., using a CCl_4_ mice model, daily administration of 40mg/kg and 80mg/kg ASTA significantly reduced plasma ALT and AST, which in turn decreased the level of liver inflammation [33]. Bhuvaneswar, S. *et al*., also demonstrated the protective effect of ASTA by supplementing 6mg/kg of ASTA in high cholesterol diets[34]. Our study coincides with the studies mentioned above, and therefore, suggesting the FFAP can be used as a material for developing healthy foods.

**Table 5:** Liver functions in all test groups.

Group	ALT (U/L)	AST (U/L)
**Normal**	11.66 ± 7.09 ^b^	7.63 ± 7.30 ^b^
**Control**	21.83 ± 4.28 ^a^	13.73 ± 5.29 ^a^
**1.6FFAP**	6.53 ± 2.46 ^c^	5.66 ± 3.49^bc^
**3.2FFAP**	4.61 ± 2.39 ^c^	2.11 ± 0.60 ^c^
**8.0FFAP**	3.73 ± 2.49 ^c^	1.93 ± 1.23 ^c^

Plasma ALT and AST from all test groups were examined to study the effect of FFAP on liver function. The result showed FFAP diets significantly decreased plasma ALT and AST, p<0.05. All values are Mean ± SD, n = 9. All data was tested using Duncan’s multiple range tests, where significance between groups (p<0.05) is denoted by dissimilar lowercase letters. Normal: Normal diet; Control: Normal diet + 0.2% cholesterol; 1.6FFAP: control diet + 1.6%FFAP; 3.2FFAP: control diet + 3.2% FFAP; 8.0FFAP: control diet + 8.0%FFAP.

### Plasma and liver antioxidant activities

**Fig 3:**
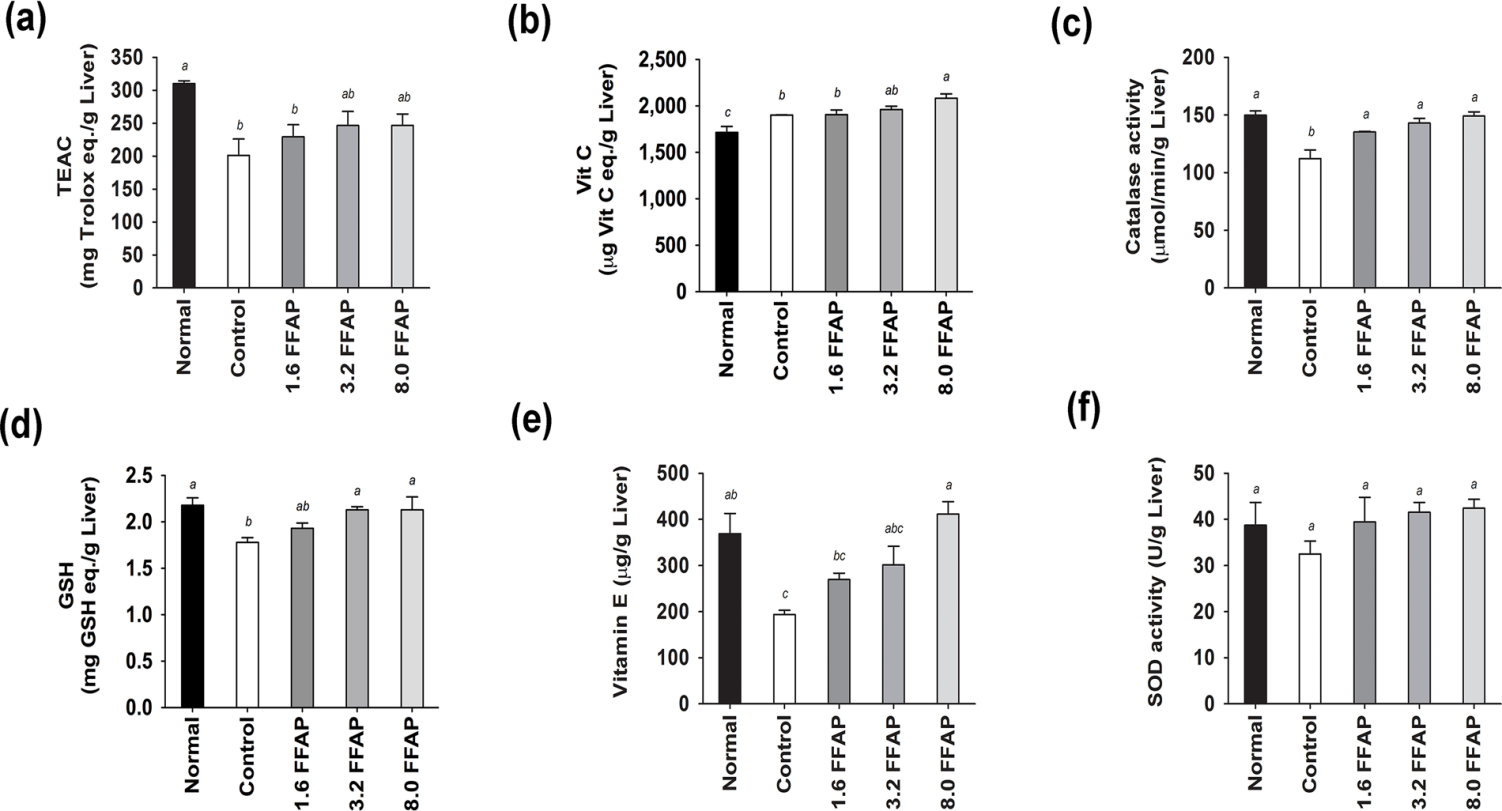
Liver anti-oxidant activities of all test groups. (a) Liver TEAC, (b) liver Vitamin C, (c) catalase activity, (d) GSH, (e) Vitamin E and (f) SOD activity. FFAP diets significantly increased liver TEAC, catalase activity, GSH and Vitamin E; and to some extent, SOD activity and plasma TEAC, compared to control, *p*<0.05. Moreover, although not significantly different, FFAP diets increased liver TEAC compared to high cholesterol control. All values are Mean ± SD, n = 9. All data were tested using Duncan’s range test where in the same column values not sharing a common letters are significantly different from one another. Normal: Normal diet; Control: Normal diet + 0.2% cholesterol; 1.6FFAP control diet + 1.6%FFAP; 3.2FFAP: control diet + 3.2% FFAP; 8.0FFAP: control diet + 8.0%FFAP.

**Fig 4:**
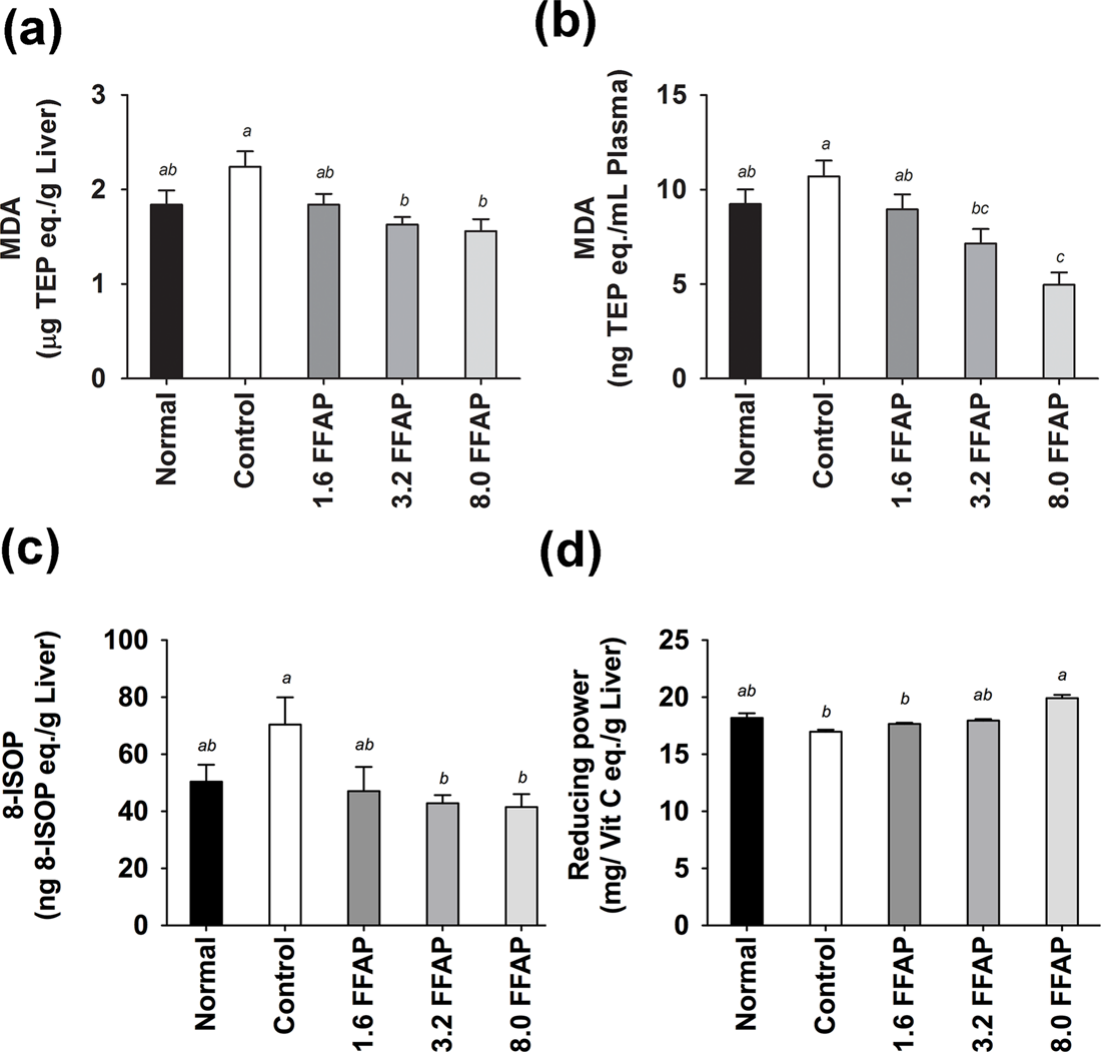
Liver and plasma MDA and 8-ISOP activities and reducing power. FFAP diets significantly decreased (a) liver MDA, (b) plasma MDA, (c) liver 8-ISOP, and significantly increased the liver reducing power (d). *p*<0.05. All values are Mean ± SD, n = 9. All data were tested using Duncan’s range test where in the same column values not sharing a common letters are significantly different from one another. Normal: Normal diet; Control: Normal diet + 0.2% cholesterol; 1.6FFAP control diet + 1.6%FFAP; 3.2FFAP: control diet + 3.2% FFAP; 8.0FFAP: control diet + 8.0%FFAP.

To investigate the effects of FFAP on antioxidant activities, we measured plasma and liver TEAC, Vitamin C and MDA; as well as liver 8-ISOP, Vitamin E, catalase, GSH and SOD from each animal. We found that the high cholesterol diet significantly decreased the levels of liver TEAC (201.32±74.56 μg Trolox eq./g Liver, Fig 3a), catalase activity (112.16±22.6 μmol/min/g Liver, Fig 3c), GSH (1.78±0.15 mg GSH eq./g Liver, Fig 3d), Vitamin E (193.46±28.48μg/g Liver, Fig 3e) levels, and to some extent, lowered the levels of liver SOD activity (32.51±8.35U/g Liver, Fig 3f), plasma TEAC (8.24±0.22 μg Trolox eq./mL, (data not shown) and plasma Vitamin C (data not shown). On the other hand, it increased liver and plasma MDA (2.24±0.49 μg TEP eq./g Liver and 10.69±2.52 μg TEP eq./dL plasma, Fig 4a and 4b; and liver 8-ISOP levels 70.41±28.67 eq./g Liver, Fig 4c). Supplementing FFAP in the high cholesterol diet was able to efficiently recover liver TEAC (between 13.89 and 22.53%, Fig 3a) and Vitamin C (1904.94±152.29 to 2082.97±142.23 μg Vit C eq./g Liver, Fig 3b) levels. It also significantly increased liver catalase activities (135.27±1.80 to 149.10±10.76 μmol/min/g Liver, Fig 3c), GSH (1.93±0.17 to 2.13±0.42 mg GSH eq./g Liver, Fig 3d) and Vitamin E levels (269.40±42.47 to 411.32±81.67 μg/g liver, Fig 3e). Moreover, it significantly reduced plasma MDA by between 16.28% and 53.60% and liver MDA by between 17.86% and 30.36% in a dose dependant manner (Fig 4a and 4b). Liver 8-ISOP was also significantly reduced by approximately 40% (Fig 4c). Furthermore, reducing power analysis showed that the liver reducing power is lower in high cholesterol diet (16.98±0.54 mg Vit C eq./g Liver), comparing to normal diet (18.18±1.24 mg Vit C eq./g liver); and the addition of 1.6, 3.2 and 8.0FFAP in the high cholesterol diet improved the reducing power to 17.66±0.33, 17.95±0.40 and 19.91±0.88 mg Vit C eq./g liver, respectively. Notably, the reducing power in 8.0FFAP diet group increased significantly by 17.26%, compared to high cholesterol control, Fig 4d.

### Correlations between plasma and liver MDA and anti-oxidant activities

MDA is a highly reactive three carbon dialdehyde produced as a by-product of polyunsaturated fatty acid peroxidation and arachidonic acid metabolism. It is usually used as an index for oxidative stress. To study the correlation of MDA with the other antioxidants after FFAP diets a Pearson correlation coefficient test was used. The correlation between MDA and anti-oxidant activities is shown in Table 6. Plasma and liver MDA was positively correlated with liver and plasma 8-ISOP (*p*<0.001). On the contrary, liver MDA is negatively correlated with plasma Vitamin C (*p*<0.05) and catalase activity (*p*<0.001).

**Table 6:** The correlations between plasma and liver malondialadhyde (MDA) and antioxidant activities of all test groups.

Antioxidant activity	MDA in blood	MDA in liver
**8-ISOP**	0.55883***	0.74836***
**Vit C**	-0.37772*	-0.1289
**SOD**	0.03713	-0.19761
**Catalase**	-0.68007***	-0.33729
**TEAC**	-0.1715	-0.39246
**GSH**	-0.27192	-0.58179*

Plasma and liver MDA is positively correlated with liver and plasma 8-ISOP (p<0.001). On the contrary, liver MDA is negatively correlated with Vitamin C (p<0.05) and catalase (p<0.001).

*p<0.05

**p<0.01

***p<0.001.

### ASTA absorption percentage and distribution

Finally, we examined the ASTA absorption efficiency in the FFAP test groups. ASTA total absorption was calculated by the formula shown below:

ASTA total absorption % = (ASTA intake (g) – ASTA output (g)) / total intake (g)×100%

Total ASTA intake was calculated using the formula: *Total ASTA intake (g) = Food intake (g) x FFAP (%) x 1% (ASTA concentration in FFAP)* and recorded in S1 Table. The result showed that the ASTA absorption in the 1.6, 3.2 and 8.0 FFAP groups were high at 78.91%, 86.56% and 86.03%, respectively; suggesting this form of ASTA powder is an effective way in promoting ASTA absorption, Table 7. Furthermore, in order to understand the distribution of ASTA in the body after it is absorbed, we also analyzed the ASTA contents in the liver, eyes and faeces from each animal. The result showed after ASTA was absorbed, it was redistributed and stored in the liver (between 0.97±0.01μg/g and 1.26±0.24μg/g) and eyes (between 10.59±3.82 μg/g and 17.21±3.53 μg/g) in a dose dependent manner. Similarly, the more ASTA that was consumed, the more the ASTA was excreted into faeces (between 303.67±8.37 μg/g and 564.82±78.59 μg/g).

**Table 7:** Astaxanthin absorption percentage and distribution.

Group	Asta absorption (%)	Liver (μg/g)	Eye (μg/g)	Fecal (μg/g)
**Normal**	ND	ND	9.09 ± 3.57^bc^	ND
**Control**	ND	ND	6.30 ± 3.35 ^c^	ND
**1.6FFAP**	78.91	0.97 ± 0.01 ^b^	10.59 ± 3.82^bc^	303.67 ± 8.37 ^b^
**3.2FFAP**	86.56	1.06 ± 0.04 ^ab^	13.00 ± 3.70 ^ab^	412.50 ± 155.61 ^ab^
**8.0FFAP**	86.03	1.26 ± 0.24 ^a^	17.21 ± 3.53 ^a^	564.82 ± 78.59 ^a^

All data was tested using Duncan’s multiple range tests, where significance between groups (p<0.05) is denoted by dissimilar lowercase letters. Normal: Normal diet; Control: Normal diet + 0.2% cholesterol; 1.6FFAP: control diet + 1.6%FFAP; 3.2FFAP: control diet + 3.2% FFAP; 8.0FFAP: control diet + 8.0%FFAP.

## Conclusion and Discussions

**Fig 5:**
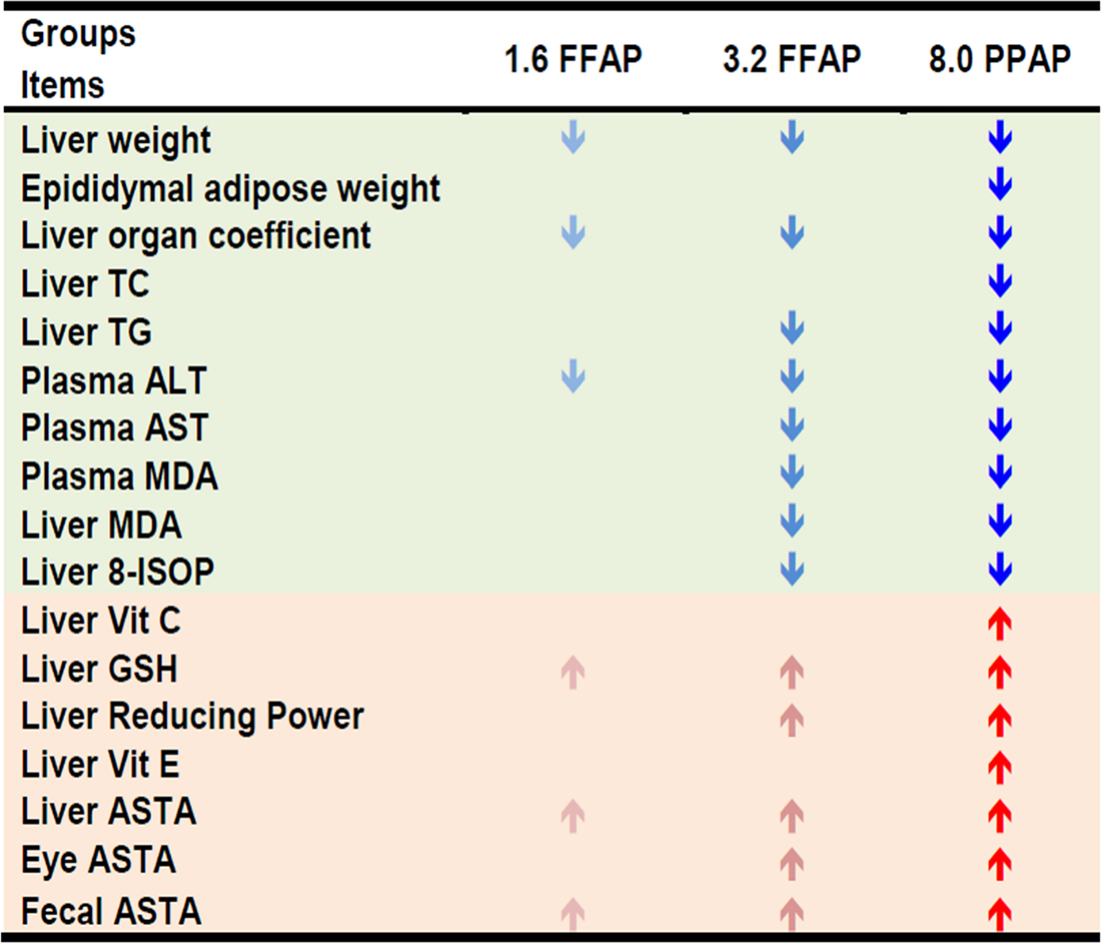
Comparison of the anti-oxidative activities and lipid metabolism between different FFAP diet groups.

In this study, ASTA was mixed with mung bean powder and made into FFAP diets and an *in vivo* hamster model was used to investigate its effects on liver metabolism and anti-oxidative activities. We found that the addition of FFAP to a high cholesterol diet significantly increased the levels of liver catalase activity, Vitamin C, Vitamin E, GSH and reducing power. Also, it significantly decreased plasma ALT and AST levels, MDA and 8-ISOP levels. Moreover, the MDA levels were positively correlated with 8-ISOP and negatively correlated to catalase, Vitamin C, Vitamin E and GSH contents. Fig 5 summarizes the overall effects on antioxidant activities and liver metabolisms of FFAP diets, from which we found the 8.0FFAP has the highest anti-oxidative activities and it effectively improves liver function and therefore could be used as a potential material in healthy food development. Our result support the findings by Shih CK *et al*., that used rats fed a high-cholesterol diet to investigate the roles of β-carotene and canthaxanthin in cholesterol metabolism and demonstrated that β-carotene and canthaxanthin are able to modulate the balance between pro-oxidation and antioxidation and inhibit cholesterol-induced oxidative stress[35]. In terms of liver lipid and fatty acid composition, we found FFAP diets efficiently deceased liver total cholesterol and triglycerides, and also increased the proportion of DHA and EPA, which are crucial constituents of Omega-3. Since Omega-3 is an important component in physiological metabolism, the increase in the ratio of DHA and EPA in the FFAP diets suggests it may have a beneficial role in these physiological processes. Indeed, research by Arnold C *et al*., demonstrated that EPA and DHA can be efficiently converted by CYP enzymes to novel epoxy and hydroxyl metabolites that are able to mediate some beneficial cardiovascular effects[36]. A study from Bell J. G. *et al*., found dietary deficiency of ASTA significantly increased the recovery of EPA and DHA in Atlantic salmon[37]. In this study, hepatocytes isolated from Atlantic salmon fed with different diets (with or without ASTA) were analyzed for anti-oxidative capacity. From the results, the authors found a significant increase in EPA and DHA when fish were fed ASTA deficient diets and suggested this may be due to a compensatory effect in order to overcome the oxidative stress caused by, for example, restricted dietary intake of antioxidant components such as Vitamin E and selenium; or inclusion of dietary pro-oxidants such as high cholesterol diets. In contrast with these findings, our study showed ASTA containing-FFAP diets increased the percentage of EPA and DHA in liver fatty acid compositions. The mechanism for this is unclear, but may be due to the differing lipid metabolic systems between salmon and hamsters. Moreover, in our study, hamsters were fed with high cholesterol diets in combination with different concentrations of FFAP. The combination of high cholesterol diet with FFAP may trigger homeostatic cellular regulatory responses to, for example, altering the activities of modulation enzymes, such as Δ6-desaturase[38, 39], Δ5-desaturase and C20 elongase, which may in turn contribute to the observed changes in fatty acid compositions.

Antioxidants are important for healthy functioning of the body. Both Vitamin C & E prevent excessive oxidative stress by reducing free radicals to stable molecules. Vitamin E has also been shown to decrease lipid peroxidation and protect unsaturated fatty acid in the phospholipids on the cell membrane. Moreover, MDA and 8-ISOP are shown to be an index for lipid peroxides[40], which are crucial factors in cell injury[41]. From our study, FFAP diets efficiently decreased oxidative stress, increased liver reducing power, catalase activities, GSH, Vitamin C and Vitamin E levels and therefore may have a beneficial health effect. Our study supports the findings by Bhuvaneswaris, S. *et al*., and Curek, G.D. *et al*., who also demonstrated that supplementing ASTA (6.0mg/kg) into high cholesterol diet significantly increased the activity of liver catalase activity[34] while 5mg/kg ASTA significantly increased liver GSH levels[42]. The ASTA absorption rates in the FFAP diet groups were high, at around 80%, suggesting the FFAP is an efficient format in improving ASTA absorption. However, we also found the amount of ASTA excreted in the faeces is proportional to its dose in the FFAP diets, which suggests that the ASTA was not entirely absorbed. The high fiber content of the FFAP formula (Fig 1) may contribute to this phenomenon as the high fiber content can promote intestinal tract peristalsis and therefore may in turn shorten the ASTA retention time. Finally, our result showed a significant amount of ASTA distributed in the liver and eyes. The redistribution of ASTA into livers and eyes is encouraging because this suggests that FFAP diet can be beneficial to certain diseases such as liver fibrosis and diabetic retinopathy. According to Yang Y *et al*., ASTA inhibits TGF-β1 induced pro-fibrogenic gene expressions, which then abolishes Smad3 phosphorylation in hepatic stellate cells and therefore attenuates liver fibrosis[43]. Moreover, a research by Dong LY et al., suggested that ASTA is able to inhibit oxidative stress and reduce apoptosis of retinal ganglion cells in db/db mice[44]. Therefore, further investigation of the beneficial roles of FFAP diets in diseases relating to liver fibrosis, such as non-alcoholic fatty liver disease (NAFLD); and diabetic retinopathy as well as its mechanisms of actions will give us further understanding of its potential applications. Taken together, our results demonstrated the FFAP diets increased liver anti-oxidative activity and reduced the concentration of lipid peroxidase and therefore it may be beneficial as a material in developing healthy food.

## Supporting Information

S1 Fig Astaxanthin powder and different diets used in this study. (A) *Free form Astaxathin powder (FFAP) used in this study and its chemical formula*. The free form astaxathin powder contains 1% pure ASTA. (B) *Images of 5 different formula diets used in this study*. From left to right: normal diet; high cholesterol control (normal diet+0.2% cholesterol); 1.6% FFAP (high cholesterol control+1.6%FFAP); 3.2% FFAP (high cholesterol control+3.2% FFAP); 8.0% FFAP (high cholesterol control+8.0%FFAP).
(PDF)

S2 Fig Food intake and body weight changes of hamsters during the 6-weeks experiment period. There were no significant differences in (A) *food intake* and (B) *body weight*, between each diet groups. All values are Mean ± SD, n = 9. All data were tested using Duncan’s range test where in the same column values not sharing a common letters are significantly different from one another. Normal: Normal diet; Control: Normal diet + 0.2% cholesterol; 1.6FFAP control diet + 1.6%FFAP; 3.2FFAP: control diet + 3.2% FFAP; 8.0FFAP: control diet + 8.0%FFAP.
(PDF)

S3 Fig The liver gross appearance and H&E staining of different diet groups. (A) *Livers gross appearance* and (B) *H&E staining* in the FFAP diet groups were similar to those in normal control. Normal: Normal diet; High Cholesterol Control: Normal diet + 0.2% cholesterol; 1.6FFAP: control diet + 1.6%FFAP; 3.2FFAP: control diet + 3.2% FFAP; 8.0FFAP: control diet + 8.0%FFAP.
(PDF)

S1 Table Astaxanthin total intake and output of all diet groups. (PDF)
