# Supplementary material for: In Vivo Effects of Free Form Astaxanthin Powder on Anti-Oxidation and Lipid Metabolism with High-Cholesterol Diet
Source: PLoS One. 2015 Aug 11;10(8):e0134733. doi: 10.1371/journal.pone.0134733 (PMC4532504; doi:10.1371/journal.pone.0134733)

S1 Fig. Astaxanthin powder and different diets used in this study

(A) *Free form Astaxathin powder (FFAP) used in this study and its chemical formula.* The free form astaxathin powder contains 1% pure ASTA.

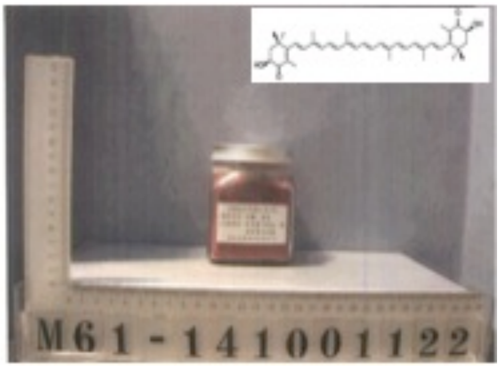

(B) *Images of 5 different formula diets used in this study.* From left to right: normal diet; high cholesterol control (normal diet+0.2 % cholesterol); 1.6% FFAP (high cholesterol control+1.6 %FFAP); 3.2% FFAP (high cholesterol control+3.2 % FFAP); 8.0% FFAP (high cholesterol control+8.0 %FFAP).

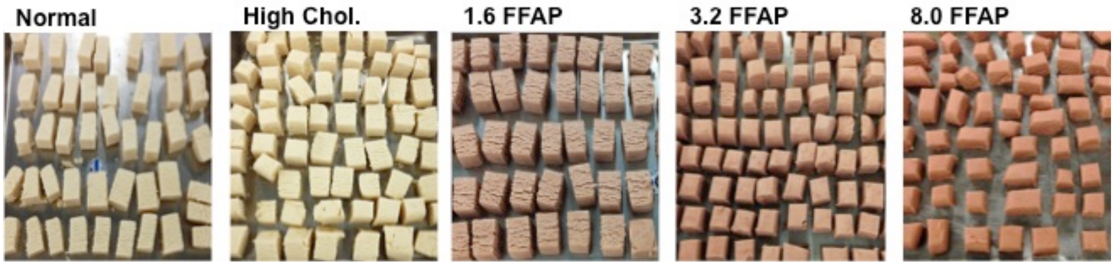

Supplement: S1 Fig — (A) Free form Astaxathin powder (FFAP) used in this study and its chemical formula. The free form astaxathin powder contains 1% pure ASTA. (B) Images of 5 different formula diets used in this study. From left to right: normal diet; high cholesterol control (normal diet+0.2% cholesterol); 1.6% FFAP (high cholesterol control+1.6%FFAP); 3.2% FFAP (high cholesterol control+3.2% FFAP); 8.0% FFAP (high cholesterol control+8.0%FFAP). (PDF) [file pone.0134733.s001.pdf]
