# Supplementary material for: In Vivo Effects of Free Form Astaxanthin Powder on Anti-Oxidation and Lipid Metabolism with High-Cholesterol Diet
Source: PLoS One. 2015 Aug 11;10(8):e0134733. doi: 10.1371/journal.pone.0134733 (PMC4532504; doi:10.1371/journal.pone.0134733)

S2 Fig. Food intake and body weight changes of hamsters during the 6-weeks experiment period. There were no significant differences in (A) *food intake* and (B) *body weight*, between each diet groups. All values are Mean ± SD, n=9. All data were tested using Duncan's range test where in the same column values not sharing a common letters are significantly different from one another. Normal: Normal diet; Control: Normal diet + 0.2 % cholesterol; 1.6FFAP control diet + 1.6 %FFAP; 3.2FFAP: control diet + 3.2 % FFAP; 8.0FFAP: control diet + 8.0 %FFAP.

(A) Food intake

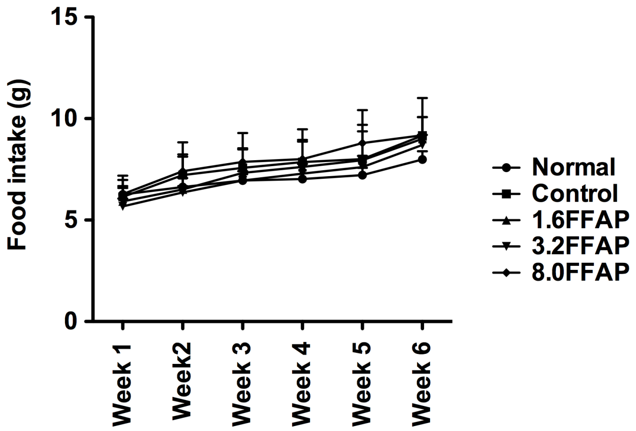

(B) Body weight

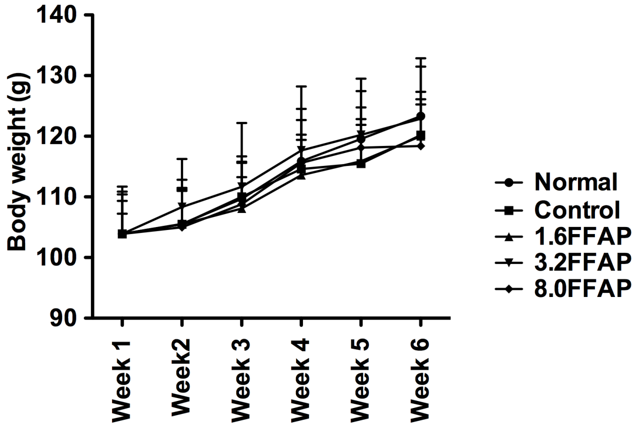

Supplement: S2 Fig — There were no significant differences in (A) food intake and (B) body weight, between each diet groups. All values are Mean ± SD, n = 9. All data were tested using Duncan’s range test where in the same column values not sharing a common letters are significantly different from one another. Normal: Normal diet; Control: Normal diet + 0.2% cholesterol; 1.6FFAP control diet + 1.6%FFAP; 3.2FFAP: control diet + 3.2% FFAP; 8.0FFAP: control diet + 8.0%FFAP. (PDF) [file pone.0134733.s002.pdf]
