# Supplementary material for: In Vivo Effects of Free Form Astaxanthin Powder on Anti-Oxidation and Lipid Metabolism with High-Cholesterol Diet
Source: PLoS One. 2015 Aug 11;10(8):e0134733. doi: 10.1371/journal.pone.0134733 (PMC4532504; doi:10.1371/journal.pone.0134733)

S3 Fig. The liver gross appearance and H&E staining of different diet groups. (A) *Livers gross appearance* and (B) *H&E staining* in the FFAP diet groups were similar to those in normal control. Normal: Normal diet; High Cholesterol Control: Normal diet + 0.2 % cholesterol; 1.6FFAP: control diet + 1.6 %FFAP; 3.2FFAP: control diet + 3.2 % FFAP; 8.0FFAP: control diet + 8.0 %FFAP.

(A) Liver gross appearance

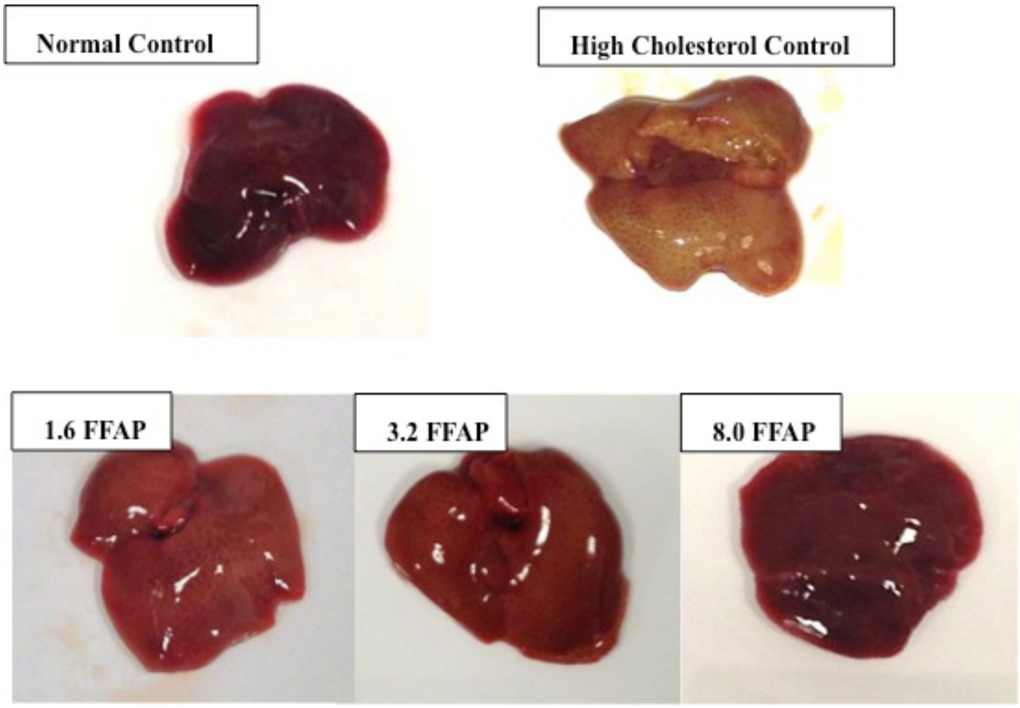

(B) H&E staining of different diet groups.

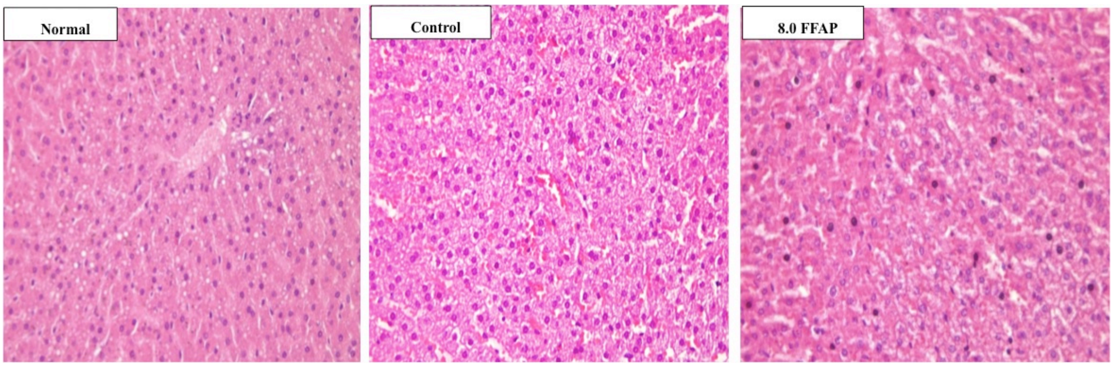

Supplement: S3 Fig — (A) Livers gross appearance and (B) H&E staining in the FFAP diet groups were similar to those in normal control. Normal: Normal diet; High Cholesterol Control: Normal diet + 0.2% cholesterol; 1.6FFAP: control diet + 1.6%FFAP; 3.2FFAP: control diet + 3.2% FFAP; 8.0FFAP: control diet + 8.0%FFAP. (PDF) [file pone.0134733.s003.pdf]
