# Supplementary material for: In Vivo Effects of Free Form Astaxanthin Powder on Anti-Oxidation and Lipid Metabolism with High-Cholesterol Diet
Source: PLoS One. 2015 Aug 11;10(8):e0134733. doi: 10.1371/journal.pone.0134733 (PMC4532504; doi:10.1371/journal.pone.0134733)
Supplement: S1 Table — (PDF) [file pone.0134733.s004.pdf]

S1 Table. Astaxanthin total intake and output of all diet groups.

Group	Astaxanthin Intake (mg/day)	Astaxanthin Output (mg/day)	Astaxanthin Total absorption (mg/day)
**Normal**	ND	ND	ND
**Control**	ND	ND	ND
**1.6 FFAP**	1.44 ± 0.17 [b]	0.30 ± 0.01 [b]	1.14 ± 0.16 [b]
**3.2 FFAP**	2.78 ± 0.16 [a]	0.37 ± 0.14 [ab]	2.41 ± 0.30 [a]
**8.0 FFAP**	3.67 ± 0.73 [a]	0.51 ± 0.04 [a]	3.16 ± 0.77 [a]

Normal: Normal diet; Control: Normal diet + 0.2 % cholesterol; 1.6FFAP: control diet + 1.6 %FFAP; 3.2FFAP: control diet + 3.2 % FFAP; 8.0FFAP: control diet + 8.0 %FFAP. All values are mean ± SD (n = 9), and in the same column values not sharing a common superscription letters are significantly different from one another by Duncan's rang test ($p < 0.05$). Total ASTA intake was calculated using the formula shown below: Total ASTA intake (g) = Food intake (g) x FFAP (%) x 1% (ASTA concentration in FFAP)
